# Analysis of *LOXL1* gene variants in Japanese patients with branch retinal vein occlusion

**Published:** 2011-12-17

**Authors:** Katsunori Hara, Masakazu Akahori, Masaki Tanito, Sachiko Kaidzu, Akihiro Ohira, Takeshi Iwata

**Affiliations:** 1 Department of Ophthalmology, Shimane University Faculty of Medicine, Izumo, Shimane, Japan; 2 National Institute of Sensory Organs, National Hospital Organization Tokyo Medical Center, Tokyo, Japan

## Abstract

**Purpose:**

Previous studies have described a possible association between exfoliation syndrome (EX) and various ocular and systemic vascular disorders; however, the association between EX and branch retinal vein occlusion (BRVO) remains unclear. Because slit-lamp examination may overlook latent deposits of exfoliation materials, an ocular biopsy is usually needed for a precise diagnosis. We evaluated a possible association between EX and BRVO using lysyl oxidase-like 1 (*LOXL1*) gene variants as alternative markers for EX.

**Methods:**

Allelic and genotypic frequencies of three *LOXL1* variants (rs1048661, rs3825942, and rs2165241) were determined for 78 consecutive Japanese patients with BRVO (11 patients with exfoliation syndrome [EX+], 67 patients without exfoliation syndrome [EX-]), and 158 patients with cataract without EX (CT) as controls.

**Results:**

The rs1048661 variant differed between the BRVO and CT groups in allelic and genotypic frequencies (p=0.0137 and p=0.0203, respectively). Subgroup analysis, compared to the CT group, showed that BRVO EX+ had significantly different allelic and genotypic frequencies of rs1048661 (p=0.00011 and p=0.000189, respectively), while BRVO EX- did not (p=0.175 and p=0.288, respectively). The frequencies of rs3825942 and rs2165241 did not differ between the BRVO and CT groups.

**Conclusions:**

No association was found between BRVO and EX if *LOXL1* variants were used as disease markers for clinically undetectable EX. The results suggested that *LOXL1* variants, well established markers for EX, are not likely genetic markers for BRVO in Japanese subjects.

## Introduction

Retinal vein occlusions (RVOs), including central retinal vein occlusion (CRVO), an occlusion at the central trunk of the retinal vein, and branch retinal vein occlusion (BRVO), an occlusion at an arteriovenous crossing where the retinal artery and vein are bound by a common adventitial sheath, are important causes of ocular morbidity [1,2]. Although CRVO and BRVO have several risk factors in common, including systemic hypertension, smoking, hyperlipidemia, and elevated plasma homocysteine [1,2], they do not fully explain the involvement of the central trunk or branch of the retinal vein circulation.

Exfoliation syndrome (EX), the most common identifiable cause of open-angle glaucoma worldwide, is an age-related, generalized disorder of the extracellular matrix characterized by the production and progressive accumulation of fibrillar extracellular material in many ocular tissues [3]. A recent genome-wide association study reported that one intronic single nucleotide polymorphism (SNP; rs2165241) and two exonic SNPs (rs1048661 [R141L], rs3825942 [G153D]) in the first exon of the lysyl oxidase-like 1 *(LOXL1)* gene on chromosome 15q24.1 are highly associated with EX in Icelandic and Swedish populations, and that none of these SNPs was associated with primary open-angle glaucoma in the two populations [4]. Several studies have confirmed the association of these SNPs with EX in other populations [5], including a Japanese population [6-11].

In addition to ocular tissues, production and progressive accumulation of exfoliation materials occur in skin and various visceral organs [3,12]. The association of EX with various systemic vascular and neurodegenerative disorders has been described in ischemic heart disease [13,14], carotid stiffness [15], cerebrovascular disease [16], Alzheimer disease [17], and hearing loss [18]. Regarding RVO, several studies have described a possible association between CRVO and EX diagnosed based on chart review [19], slit-lamp examination [20], histopathologic studies in enucleated eyes [21,22], and a combination of slit-lamp examination and conjunctival biopsy [23], while only a few studies have evaluated the association between BRVO and EX [19,20]. Recently, the role of the *LOXL1* polymorphism has been tested in several ocular [24] and systemic [25,26] pathologies to explore the association between EX and these pathologies, suggesting the usefulness of analyzing *LOXL1* variants as a disease marker for EX.

In the current study, we tested the association between *LOXL1* variants and BRVO in a Japanese population to explore a possible association between EX and BRVO.

## Methods

### Subjects

Unrelated Japanese subjects with BRVO (n=78) were consecutively recruited at the Shimane University Hospital and Iinan Hospital in Shimane, Japan. The BRVO group was divided into two subgroups based on the presence (EX+, n=11) or absence (EX-, n=67) of clinically detectable ocular deposits of exfoliation material. The data set from patients with cataract without deposits of exfoliation material (CT, n=158) reported in our previous study [11] served as a control. The demographic data including age and gender for each group are summarized in Table 1.

**Table 1:** Summary of study populations.

** **	**BRVO**			** **	** **
	**Total**	**EX-**	**EX+**	**CT**	**p-value**
No. of subjets	78	67	11	158	
**Men/Women**					
No.	32/46	29/38	3/8	45/113	0.0568*
%	41/59	33/67	27/73	28/72	
**Age (years)**					
Mean±SD	73.2±9.6	72±9.4	80.5±6.8	76.9±4.9	5.81×10^−5^†
Range	47–88	47–87	69–88	70–90	

*Fisher’s exact probability test between BRVO (total) and CT groups. †Unpaired *t*-test between BRVO (total) and CT groups.

### Methods

The current study adhered to the tenets of the Declaration of Helsinki. The institutional review boards of both hospitals reviewed and approved the research. All subjects provided written informed consent. All subjects underwent a dilated pupil examination of the anterior segments, ocular media, and fundus using a slit-lamp (RO5000, Buchmann Deutschland, Düsseldorf, Germany) and a funduscope (BS-III, Neitz Instruments, Tokyo, Japan). BRVO was diagnosed if the fundus examination revealed venous dilation and tortuosity with flame-shaped and dot-blot hemorrhages in a wedge-shaped region. Patients with CRVO and hemi-CRVO were excluded. Deposits of exfoliation material were identified if the slit-lamp examination revealed a typical pattern of exfoliation material on the anterior lens surface and/or pupillary margin.

### DNA genotyping

Genomic DNA was extracted from the peripheral white blood cells of each subject. A polymerase chain reaction was performed using primers designed to amplify the genomic region containing both rs1048661 and rs3825942 (forward primer: 5′-AGG TGT ACA GCT TGC TCA ACT C-3′ and reverse primer: 5′-TAG TAC ACG AAA CCC TGG TCG T-3′) or only rs2165241 (forward primer: 5′-AGA ATG CAA GAC CTC AGC ATG AG-3′ and reverse primer: 5′-TAG TGG CCA GAG GTC TGC TAA G-3′). The sequence was determined based on the dideoxy terminator method using an ABI PRISM 3130xl Genetic Analyzer (Applied Biosystems, Foster City, CA) according to the manufacturer’s protocol. We used SeqScape Software version 2.5 (Applied Biosystems) to analyze the sequence alignment.

### Statistical analysis

Statistical analysis was performed using R version 2.6.2. Fisher’s exact test was used to compare the allele or genotype frequencies of each group with the controls.

## Results

The allelic and genotypic counts and frequencies of SNPs rs1048661, rs3825942, and rs2165241 within *LOXL1* are shown in Table 2. Compared to the CT group, the T allele and TT genotype frequencies of rs1048661 were higher in patients with BRVO (p=0.0137 and p=0.0203, respectively). In subgroup analysis, compared to the CT group, the group with BRVO with exfoliation material deposits (EX+) had significantly different allelic and genotypic frequencies (p=0.00011 and p=0.000189, respectively), while the group with BRVO without exfoliation material deposits (EX-) had no difference in allelic and genotypic frequencies (p=0.175 and p=0.288, respectively). Compared to the CT group, the frequencies of the G allele of rs3825942 and the C allele of rs2165241 were higher in the BRVO EX+ groups with borderline significance (p=0.0933 and p=0.0908, respectively), but the allelic and genotypic frequencies did not differ between any pairs of BRVO total or BRVO EX- and the CT group.

**Table 2:** Allelic and genotypic counts and frequencies of SNPs rs1048661, rs3825942, and rs2165241.

** **	**BRVO**						** **	** **	** **	** **	** **
** **	**Total**		**EX-**		**EX+**		**CT**		p-value*		
** **	**Count**	**Frequency**	**Count**	**Frequency**	**Count**	**Frequency**	**Count**	**Frequency**	**Total versus CT**	**BRVO EX- versus CT**	**EX+ versus CT**
rs1048661											
**Allele**											
T	86	0.566	67	0.515	19	0.864	140	0.443	0.0137	0.175	1.10×10^−4^
G	66	0.434	63	0.485	3	0.136	176	0.557	** **	** **	** **
**Genotype**											
TT	24	0.316	16	0.246	8	0.727	25	0.158	0.0203	0.288	1.89×10^−4^
TG	38	0.500	35	0.538	3	0.273	90	0.570	** **	** **	** **
GG	14	0.184	14	0.215	0	0	43	0.272	** **	** **	** **
rs3825942											
**Allele**											
G	131	0.862	110	0.846	21	0.955	255	0.807	0.155	0.348	0.0933
A	21	0.138	20	0.154	1	0.045	61	0.193	** **	** **	** **
**Genotype**											
GG	57	0.750	47	0.723	10	0.909	101	0.639	0.212	0.424	0.209
AG	17	0.224	16	0.246	1	0.091	53	0.335	** **	** **	** **
AA	2	0.026	2	0.031	0	0	4	0.025	** **	** **	** **
rs2165241											
**Allele**											
C	135	0.877	113	0.856	22	1.000	277	0.877	1	0.541	0.0908
T	19	0.123	19	0.144	0	0	39	0.123	** **	** **	** **
**Genotype**											
CC	61	0.792	50	0.758	11	1.000	123	0.778	0.765	0.685	0.335
CT	13	0.169	13	0.197	0	0	31	0.196	** **	** **	** **
TT	3	0.039	3	0.045	0	0	4	0.025	** **	** **	** **

*Fisher's exact probability test.

## Discussion

To the best of our knowledge, this is the first study to identify a possible association between *LOXL1* variants and BRVO. The prevalence of clinical EX increases with age, especially after age 60 [3]. Accordingly, detection of exfoliation material deposits by slit-lamp examination may overlook latent EX. Indeed, previous studies have suggested that the prevalence of exfoliation material deposits found on histopathologic assessment of ocular specimens was roughly double compared with the slit-lamp examination [27,28]. A conjunctival biopsy can detect preclinical EX that is not evident on slit-lamp examination [23]; however, because the biopsy is invasive, it cannot be used for all patients. The role of the *LOXL1* polymorphism has been tested in several pathologies including wet and dry age-related macular degeneration and polypoidal choroidal vasculopathy in a Japanese population [24], Alzheimer disease in a Swedish population [25], and cardiovascular disease in a Hungarian population [26]. Fuse et al. found a significant association between the rs1048661 polymorphism and wet age-related macular degeneration in a Japanese population [24]. These studies encouraged us to use the *LOXL1* polymorphism as an alternative marker of clinically undetectable EX other than invasive biopsy/histopathology.

Among the three SNPs reported [4], rs1048661 has been consistently suggested as the most significant indicator of EX/glaucoma in Icelandic, Swedish, and Japanese populations [6-11]. Accordingly, our results of a significant difference in allelic and genotypic frequencies of rs1048661 between all subjects with BRVO and CT or BRVO EX+ and CT groups confirmed previous observations of the strong role of this SNP in EX. The results also suggested that using this SNP, we can detect a case–control association for EX even with such a small number of subjects (n=11) in a case group. In the same context, the other two SNPs, which showed only a borderline difference between BRVO EX+ and CT groups, may not have enough discriminatory power with this small number of subjects.

Since both the BRVO EX- and CT groups, which were classified based on slit-lamp examination as not having EX, were identical except for the presence or absence of BRVO, comparison between these two groups should provide the most reliable information about the possible role of the *LOXL1* variants in BRVO. As a result, the significant difference observed in rs1048661 between the case and control groups was canceled in the analyses between the BRVO EX- and CT groups, suggesting that the percentage of the population at risk of EX is not significantly higher in the BRVO group. A retrospective chart review reported exfoliation material deposits in 6.0% of eyes with BRVO and 6.9% of eyes with CRVO [19], suggesting a lesser extent of BRVO than CRVO in these subjects, since the BRVO/CRVO ratio was 3.2 in the general population [29]. By clinical observation of consecutive cases, the prevalence rates of EX were 8.2% in eyes with BRVO and 20.8% in eyes with CRVO compared with 5.2% in control eyes; thus the authors concluded that EX is likely a risk factor for CRVO [20]. A retrospective chart review showed that RVO occurs more frequently in eyes more affected by EX, and that the most frequent type of RVO that occurred in EX was CRVO (50%) followed by about half that prevalence of BRVO (28%) [23]. In this study, the prevalence rate of EX was 14% in eyes with BRVO from consecutive cases, which may be higher than the rate of EX in BRVO cases and normal control subjects in previous reports [19,20]. Differences in the race or age of subjects may explain the discrepancy, but this needs to be clarified. Taken together with previous studies, our results suggest that there is no direct role of *LOXL1* variants or EX in the development of BRVO in our Japanese subjects.

In summary, we tested the possible association of *LOXL1* variants with BRVO. We did not find an association between BRVO and EX if the *LOXL1* variants were used as disease markers for clinically undetectable EX.
